# Personalized Evaluation of Biomarker Value: A Cost-Benefit Perspective

**DOI:** 10.1007/s12561-014-9122-4

**Published:** 2014-11-21

**Authors:** Ying Huang, Eric Laber

**Affiliations:** 1 Biostat & Biomath Program, Fred Hutchinson Cancer Center, Seattle, WA 98109 USA; 2 Department of Statistics, North Carolina State University, 2311 Stinson Drive, Raleigh, NC 27695 USA

**Keywords:** Adaptive bootstrap, Biomarker, Cost-benefit, Semiparametric location-scale model, Subject-specific expected benefit, Treatment selection

## Abstract

For a patient who is facing a treatment decision, the added value of information provided by a biomarker depends on the individual patient’s expected response to treatment with and without the biomarker, as well as his/her tolerance of disease and treatment harm. However, individualized estimators of the value of a biomarker are lacking. We propose a new graphical tool named the subject-specific expected benefit curve for quantifying the personalized value of a biomarker in aiding a treatment decision. We develop semiparametric estimators for two general settings: (i) when biomarker data are available from a randomized trial; and (ii) when biomarker data are available from a cohort or a cross-sectional study, together with external information about a multiplicative treatment effect. We also develop adaptive bootstrap confidence intervals for consistent inference in the presence of nonregularity. The proposed method is used to evaluate the individualized value of the serum creatinine marker in informing treatment decisions for the prevention of renal artery stenosis.

## Introduction

Advances in lab technology have led to the discovery of a large number of biomarkers and medical tests that are potentially useful for guiding treatment decisions. However, these biomarkers and tests may be costly, invasive, or otherwise burdensome. Examples include the Oncotype Dx test for predicting the response to adjuvant chemotherapy, which currently costs around $4,000 USD (http://www.breastcancer.org/symptoms/testing/types/oncotype_dx), and Amniocentesis in pregnant women for detecting chromosomal abnormality, which is associated with a risk of miscarriage. Therefore, when a biomarker or test is optional, patients and clinicians must determine whether the biomarker provides useful information for treatment selection beyond readily available information such as patient demographics and standard test results. Hereafter, we use the term “covariate” for any other readily available patient information.

To make an informed decision regarding whether to have a biomarker-based diagnostic test, it is important to understand how the test affects a patient’s clinical consequence. In particular, one needs to quantify the impact of the additional marker using a metric that is intuitive and interpretable to patients. The metric should also flexibly account for individual differences to facilitate personalized decision making. Classical statistical measures for biomarker evaluation are not satisfactory for this purpose. For example, common measures of classification performance like sensitivity, specificity, and the receiver operating characteristics curve [22, 35] are not directly relevant for individual patients’ decision making because they are defined conditional on disease status and do not reflect an individual’s risk of disease. Common measures of risk prediction performance like the predictiveness curve [16, 23] and positive and negative predictive values [1] are informative about disease risk, but by themselves do not account for other important information for decision making such as the effects of available treatments on the targeted disease and additional treatment costs (e.g., the side effect and the monetary cost). Many other recent measures of a marker’s value in a treatment decision, e.g., the overall reduction in the disease rate through treatment selection [27, 31–34], the conditional distribution of the risk difference between comparative interventions in a population [7, 14, 18] and in the marker-positive group [9, 34], focus on the marker’s average effect on the entire population only with respect to the burden of the targeted disease.

There are multiple factors affecting a patient’s clinical consequence, including the patient’s expected disease outcome with or without treatment and the patient’s tolerance of the disease harm and the treatment harm. Decision theory provides an appealing framework for incorporating these cost-benefit considerations into model evaluation and has received much attention in recent years. Examples include the decision curve analysis developed for characterizing the performance of disease risk prediction models [2, 29] and the net benefit for characterizing treatment effect prediction models [4, 25, 30]. Nevertheless, existing work in this paradigm focuses primarily on quantifying the average value of a marker to the whole population for guiding individualized treatment selection instead of allowing the evaluation of the marker value itself to be individualized. For example, the decision curve quantifies a risk prediction model’s utility in units of the benefit for a true positive, which does not account for patient-specific treatment effects. The net benefit quantifies the utility of a marker-based treatment-selection strategy in units of the disease harm, which is intuitive and easy to communicate to patients and clinicians. The utility, however, is measured relative to the strategy of no treatment, which may not be a patient’s best choice based on the available covariate information.

In this paper, we propose a new statistical measure, the subject-specific expected benefit curve, for characterizing the additional value of a biomarker to individual patients from a cost-benefit perspective. As will be shown in Sect. 2, it is built upon an existing decision-theoretic framework [17, 25, 30] but is expanded to allow individualized evaluation of a marker by accounting for individual differences in treatment effect on disease and in tolerance of disease and treatment harm. In particular, this curve quantifies the personalized expected benefit of measuring a marker, which is defined as the reduction in the minimum total disease and treatment cost by incorporating the marker information into an individual’s treatment decision. The points on the curve are defined conditional on an individual’s baseline covariates and one’s choice of treatment/disease cost ratio. We develop semiparametric estimators for this measure that are broadly applicable to both randomized trials and cohort or cross-sectional studies. We also develop adaptive bootstrap confidence intervals that are consistent even in the nonregular setting in which a subject’s specific treatment/disease cost ratio coincides with one’s expected treatment effect on disease outcome.

In Sect. 2, we introduce the concept of the subject-specific expected benefit curve. We develop estimation methods and theoretical results for both randomized trials and cohort studies. We examine finite sample performance of the proposed estimators using simulation studies in Sect. 3. In Sect. 4, we apply the proposed methodology to demonstrate the personalized evaluation of serum creatinine as a biomarker for guiding treatment decision in preventing renal stenosis. Concluding remarks are made in Sect. 5.

## Method

Let $D$ denote an undesirable binary outcome that we call “disease,” coded 0 for non-diseased and 1 for diseased. Assume that there is a treatment available, and let $A=1, 0$ indicate the decision or recommendation for a subject to have or not have the treatment based on some model. Let ${X}$ denote baseline subject covariates, possibly multivariate, which can be used to inform the treatment decision. Suppose there is a biomarker or a marker combination $Y$ that, if measured, has the potential to further inform the treatment decision. If the marker is costly to measure, the decision to collect the marker should be informed by an estimate of the *individual-specific* benefit offered by the marker. In our motivating example of serum creatinine evaluation, a female subject must decide whether to use renal angioplasty to prevent renal stenosis. A model based on her baseline characteristics $X$, such as age, body mass index, smoking status, etc., can be used to predict the probability of having renal stenosis with or without the treatment. Based on this prediction, she can then incorporate her tolerance of the side effects of renal angioplasty to make a treatment decision. If she was also offered the option of having a lab test measuring serum creatinine level, $Y$, she should only opt to have the lab test if the predictive model incorporating both $Y$ and $X$ performs significantly better than the model using $X$ alone so as to justify the cost of the test.

We propose to quantify the individual-specific value of a biomarker with the expected reduction in an individual’s total disease and treatment cost as the result of measuring the biomarker, under the assumption that an individual would make the optimal treatment decision based on available information either before or after the biomarker measurement. To put the disease cost and the treatment cost on a common scale, we follow the decision-theoretic framework of [30] by allowing each individual to input a ratio of the cost per treatment relative to the cost per disease event, namely $\delta$, which reflects the individual’s tolerance of the treatment burden relative to his/her tolerance of the disease burden.

Define the *cost* per targeted disease event as one so that the total disease and treatment cost will be represented in units of the burden per disease event. For a subject with covariate $X=x$, this total cost, given a treatment decision rule $A$ that is a function of either $X$ alone or $X$ plus $Y$, can be computed as follows.

Let $D(1)$ and $D(0)$ indicate the potential disease outcome if a subject does or does not receive the treatment. First, if a subject with covariates $X=x$ makes a treatment decision based on $X$ alone, i.e., $A=A(X)$, then his/her expected disease rate if receiving/not receiving treatment according to decision rule $A(x)$ would equal $\sum _{a=0}^1 I\{A(x)=a\}E\{D(a)|X=x\}$, where $I()$ is the indicator function; the corresponding cost of treatment is $\delta \times A(x)$ since the cost of treatment will only be incurred when $A(x)=1$. As a result, the total disease and treatment cost based on $A(X)$ equals

1 $$\begin{aligned} \sum _{a=0}^1I\{A(x)=a\}E\{D(a)|X=x\}+\delta \times A(x). \end{aligned}$$

The optimal treatment-selection rule, say $A^{\mathrm {opt}}(x)$, that minimizes (1) is

$$\begin{aligned} A^{\mathrm {opt}}(x) = \mathrm{argmin}_{a}E\{D(a)|X=x\}+\delta \times a = I\{\Delta (x)>\delta \}, \end{aligned}$$

where $\Delta (x)=E\{D(0)|X=x\}-E\{D(1)|X=x\}$ is the risk difference between non-treated and treated conditional on $X=x$. That is, a subject will choose to treat the disease only if one’s risk difference is greater than the cost ratio $\delta$ and will not treat otherwise. Equivalently, $\delta$ is the risk difference at which the treatment benefit (i.e., the risk difference for disease) exactly compensates for harm of treatment [30]. The total disease and treatment cost given $A^{\mathrm {opt}}(x)$ equals

2 $$\begin{aligned} \mathrm{Cost}_x^1(\delta )=E\{D(0)|X=x\}-[{ \Delta (x)-\delta }]_{+} , \end{aligned}$$

where $[u]_{+}=max(0,u)$ is the positive-part function.

Second, if a subject with $X=x$ makes a treatment decision based on both covariates and the additional marker, i.e., $A=A(X,Y)$, then the total disease and treatment cost is

3 $$\begin{aligned}&P\{A(X,Y)= 0|X=x\}\times E\{D(0)|A(X,Y)=0,X=x\}\nonumber \\&\qquad +\,P\{A(X,Y)=1|X=x\}\times E\{D(1)|A(X,Y)=1,X=x\}&\nonumber \\&\qquad +\,\delta \times P\{A(X,Y)=1|X=x\}&\nonumber \\&\quad = E\left[ D(0)\times I\{A(X,Y)=0\}|X=x\right] \nonumber \\&\qquad +\,E\left[ D(1)\times I\{A(X,Y)=1\}|X=x\right] +\delta \times E\{A(X,Y)|X=x\}&\nonumber \\&\quad = E\{D(0)|X=x\} - E\{D(0)\times A(X,Y)|X=x\}+E\{D(1)\times A(X,Y)|X=x\}\nonumber \\&\qquad +\,\delta \times E\{A(X,Y)|X=x\}&\nonumber \\&\quad = E\{D(0)|X=x\} - E \left\{ A(X,Y)\times \left[ E \{D(0)|Y,X\}- E\{D(1)|Y,X\}\right] |X=x\right\} \nonumber \\&\qquad +\,\delta \times E\{A(X,Y)|X=x\}&\nonumber \\&\quad = E\{D(0)|X=x\}-E\left[ A(X,Y)\times \{\Delta (X,Y)-\delta \}|X=x\right] . \end{aligned}$$

It follows that the treatment-selection rule that minimizes (3), say $A^{\mathrm {opt}}(x,y)$, is

$$\begin{aligned} A^{\mathrm {opt}}(x, y) = \mathrm{argmin}_{a}-a\times \{\Delta (x,y)-\delta \} = I\{\Delta (x,y)>\delta \}, \end{aligned}$$

where $\Delta (x,y)=E\{D(0)|X=x,Y=y\}-E\{D(1)|X=x,Y=y\}$ is the risk difference between non-treated and treated conditional on $X=x$ and $Y=y$. Corresponding total cost due to either disease or treatment for a subject with $X=x$ equals

4 $$\begin{aligned} \mathrm{Cost}_x^2(\delta )=E\{D(0)|X=x\}-E\left[ \{\Delta (X,Y)-\delta \}_{+}|X=x\right] . \end{aligned}$$

We define the expected benefit for a subject with covariates $X=x$ and cost ratio $\delta$ as the reduction in the minimum total cost by adding marker $Y$ into the disease risk model conditional on $X$:

5 $$\begin{aligned} \mathrm{EB}_x(\delta )=\mathrm{Cost}_x^1(\delta )-\mathrm{Cost}_x^2(\delta )=E\left[ \{\Delta (X,Y)-\delta \}_{+}|X=x\right] -[\Delta (x)-\delta ]_{+}. \end{aligned}$$

**Fig. 1:**
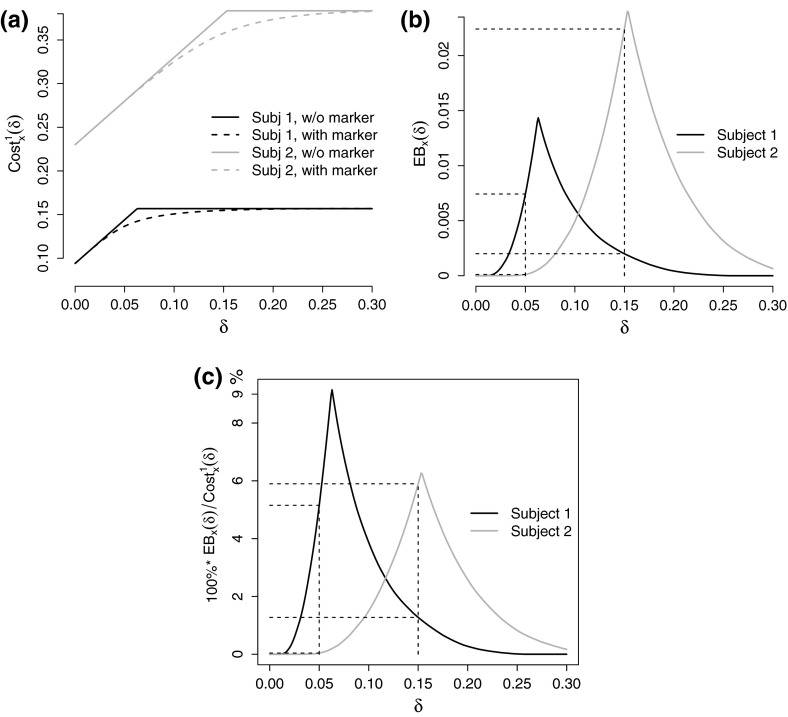
Subject-specific cost curves (**a**), expected benefit curves (**b**), and curves of relative reduction in cost by measuring serum creatinine in guiding the treatment of stenosis (**c**)

The subject-specific expected benefit curve is defined as the curve of $\mathrm{EB}_x(\delta )$ versus $\delta$. One can also plot $\mathrm{EB}_x(\delta )/\mathrm{Cost}_x^1(\delta )$ versus $\delta$ to show the relative reduction in cost by measuring $Y$. Examples of the curves of $\mathrm{Cost}_x^1(\delta )$ and $\mathrm{Cost}_x^2(\delta )$ for different $x$ levels are displayed in Fig. 1a, which will be discussed later in the data example. Corresponding curves of $\mathrm{EB}_x(\delta )$ and $\mathrm{EB}_x(\delta )/\mathrm{Cost}_x^1(\delta )$ versus $\delta$ are displayed in Fig. 1b and c. Note that a change-point exists in the curve of $\mathrm{Cost}_x^1(\delta )$ versus $\delta$ and in the subject-specific expected benefit curves, where there is a dramatic change in the shape of the curve. This point corresponds to $\delta =\Delta (x)$, the treatment effect conditional on $X=x$, which is the specific cost ratio where a subject’s optimal treatment absent biomarker $Y$ changes.

### Estimation

In this section, we consider estimation of $\mathrm{EB}_x(\delta )$ and $\mathrm{EB}_x(\delta )/\mathrm{Cost}_x^1(\delta )$, the subject-specific expected benefit on absolute and relative scales. We develop semiparametric estimation methods for data either from a randomized trial or from a cohort or cross-sectional study.

#### Estimation with Data from a Randomized Trial

Biomarker studies nested within randomized trials are natural sources for estimating the subject-specific expected benefit curve. Let $T=0,1$ indicate the assignment to the untreated and the treated arm respectively in a two-arm randomized trial. Suppose we observe i.i.d. samples of $(Y_i, X_i, T_i, D_i), i=1,\ldots , n$, one observation for every participant in the trial. The estimation of cost and expected benefit consists of two steps: (i) estimation of the disease risk conditional on $T,X,Y$ and (ii) estimation of the distribution of $Y$ conditional on $X$.

The task in the first step is to estimate $E\{D(0)|X,Y\}$ and $\Delta (X,Y)$. We make the following assumptions: (i) stable unit treatment value assumption (SUTVA) and consistency: {D(0),D(1)} of one subject is independent of the treatment assignments of other subjects, and the observed outcome is equal to the potential outcome under treatment actually received; (ii) ignorable treatment assignments assumption: $T\perp D(0),D(1) | X,Y$. Assumption (i) is plausible in trials where participants do not interact with one another, and assumption (ii) is ensured by randomization. Under these assumptions, $E\{D(t)|X,Y\}=E\{D(t)|X,Y,T\}=E(D|X,Y,T=t)$ for $t=0,1$, and $\Delta (X,Y)=E(D|X,Y,T=0)-E(D|X,Y,T=1)$. Thus, $E\{D(0)|X,Y\}$ and $\Delta (X,Y)$ can be estimated by modeling the disease risk as a function of $T$, $X$, and $Y$. We adopt a generalized linear model (GLM)

$$\begin{aligned} g \{P(D=1|{X},Y,T)\}=\theta _0+\theta _1T+\theta _2^{\prime }{X}+\theta _3Y+\theta _4^{\prime }{X}T+ \theta _5YT, \end{aligned}$$

where $g$ is a link function such as logit link or inverse normal-CDF link. We derive $\hat{\theta }$ as the maximum likelihood estimator (MLE) of $\theta$.

In the second step, there are many options to estimate the distribution of $Y$ conditional on $X$. Here we adopt a semiparametric location-scale model [12] for the distribution of $Y$ conditional on $X=x$: $Y_i=\mu (X_i)+\sigma (X_i)\epsilon _i$ and $F_0(s)=P(\epsilon \le s)$, such that $F_{x}(y)=P(Y \le y|X=x)=F_{0}\left[ {\left\{ y-\mu (x)\right\} }/{\sigma (x)}\right]$, where $F_0$ is some baseline residual distribution with mean 0 and variance 1, $\mu (X)$ and $\sigma (X)$ are the location and scale functions, respectively.

Let $\mu (x)=\gamma ^{\prime }U(x)$ and $\log \{\sigma (x)\}=\eta ^{\prime }W(x)$ with $U(x)$ and $W(x)$ being specified functions of $x$. For example, for binary $X$, $U(X)$ and $W(X)$ could be $(1,X)$, while for continuous $X$, $U(X)$ and $W(X)$ could be a B-spline basis for $X$. As in [12], we use the following estimating equations for $\gamma$ and $\eta$:

$$\begin{aligned} \sum ^{n}_{i=1}\left\{ \frac{\partial }{\partial \gamma }\mu (X_i)\right\} \frac{Y_{i}-\mu (X_i)}{\text{ var }(Y_i)}=0,\\ \sum ^{n}_{i=1}\left\{ \frac{\partial }{\partial \eta }\sigma ^2(X_i)\right\} \frac{(Y_{i}-\mu (X_i))^{2}-\sigma ^{2}(X_{i})}{ \text{ var }\left[ \{Y_i-\mu (X_i)\}^2\right] }=0. \end{aligned}$$

Write $U_{i}=U(X_{i})$ and $W_{i}=W(X_{i})$, then under the Gaussian higher moment relationship $\text{ var }\left[ \left\{ Y_i-\mu (X_i)\right\} ^2\right] =2\text{ var }(Y_i)^2$ [12], the above estimating equations equal

$$\begin{aligned} \sum ^{n}_{i=1}U_{i}(Y_{i}-\gamma ^{\prime }U_{i})/\sigma ^{2}(X_{i})=0,\\ \sum ^{n}_{i=1}W_{i}\left\{ (Y_{i}-\gamma ^{\prime }U_{i})^{2}-\sigma ^{2}(X_{i})\right\} / \sigma ^{2}(X_{i})=0, \end{aligned}$$

from which we can solve for $\hat{\gamma }$ and $\hat{\eta }$. We then estimate $F_0$ empirically using residuals $e_i= \left\{ {Y_i-\hat{\gamma }^{\prime }U_i}\right\} /{\exp \left( \hat{\eta }^{\prime }W_i\right) },i=1,\ldots ,n$.

Finally, note that in (2) and (5), $\Delta (x)$ can be represented as $E_{Y|x}\{\Delta (x,Y)\}$, the expectation of $\Delta (X,Y)$ conditional on $X=x$. Thus, we estimate $\mathrm{Cost}_{x}^1(\delta )$ in (2) and $\mathrm{EB}_x(\delta )$ in (5) with

6 $$\begin{aligned} \widehat{\mathrm{Cost}}_{x}^1(\delta )&= \frac{1}{n}\sum _{i=1}^n\widehat{\mathrm{Risk}}\left\{ x,Y_i^{\star }(x)\right\} -\left[ \frac{1}{n}\sum _{i=1}^n\hat{\Delta }\left\{ x,Y_i^{\star }(x)\right\} -\delta \right] _{+},\nonumber \\ \widehat{\mathrm{EB}}_{x}(\delta )&= \frac{1}{n}\sum _{i=1}^n\left[ \hat{\Delta }\left\{ x,Y_i^{\star }(x)\right\} -\delta \right] _{+}-\left[ \frac{1}{n}\sum _{i=1}^n\hat{\Delta }\left\{ x,Y_i^{\star }(x)\right\} -\delta \right] _{+}, \end{aligned}$$

where $Y^{\star }_{i}(x)=\hat{\gamma }^{\prime }U(x)+\exp \left\{ \hat{\eta }^{\prime }W(x)\right\} e_{i}$, $\widehat{\mathrm{Risk}}(X,Y)=\hat{P}(D=1|T=0,X,Y)$ is the estimated risk of disease, and $\widehat{\Delta }(X,Y)$ is the estimated risk difference between untreated and treated conditional on $X$ and $Y$ based on MLE $\hat{\theta }$.

#### Estimation with Data from a Cohort or Cross-sectional Study

Under the SUTVA, consistency, and ignorable treatment assignments assumptions, the subject-specific expected benefit curve can also be derived when we have biomarker data from a cohort or a cross-sectional study that can be used to estimate risk prediction models, if in addition we have external information about a multiplicative treatment effect. The latter can be obtained, e.g., from historical data or from a different study. The assumption of a relative risk $rr \in (0,1)$ for treated versus untreated has frequently been made in evaluating the use of risk models for recommending therapy, including the Gail model for advising the use of tamoxifen to prevent breast cancer [3, 10, 11]. It implicitly requires a monotone treatment effect, i.e., in terms of the disease, the treatment will not cause harm [17]. This multiplicative treatment effect assumption implies a one-to-one correspondence between the risk of disease in the population and the risk difference between untreated and treated, i.e., $\Delta (X,Y)=\mathrm{Risk}(X,Y)\times (1-rr)$, where $\mathrm{Risk}(X,Y)=P\{D(0)=1|X,Y\}$. In other words, in this scenario, we can interpret the subject-specific expected benefit for an individual as the reduction in the total disease and treatment cost by measuring the biomarker, under the condition that a subject with a treatment/disease cost ratio $\delta$ opts to take treatment whenever the predicted disease risk is greater than a threshold value of $\delta /(1-rr)$.

Estimation of $\mathrm{Cost}_x^1(\delta )$ and $\mathrm{EB}_x(\delta )$ again consists of two steps. In the first step, we model the disease risk as a function of $X$ and $Y$ using a GLM $g\{P(D=1|X,Y)\}=\theta _0+\theta _1^{\prime }X+\theta _2Y$. In the second step, we model the distribution of $Y$ conditional on $X$ using a semiparametric location-scale model as in Sect. 2.1.1. Finally, we estimate $\mathrm{Cost}_{x}^1(\delta )$ and $\mathrm{EB}_x(\delta )$ as in (6), where we estimate $\hat{\Delta }(x,y)$ with $\widehat{\mathrm{Risk}}(x,y)\times (1-rr)$.

We note that the proposed estimation procedures can be easily extended to allow for subsampling of $Y$, e.g., when the biomarker is only measured from a case-control sample nested within a randomized trial or a cohort study. Let $p_i$ be the probability of sampling $Y$ from the $i^\mathrm{th}$ subject in the study, we can estimate the subject-specific expected benefit curve by weighting the contribution of subject $i$ inversely by $p_i$ when estimating both the risk model and the conditional biomarker distribution.

Note that in the special case where $X$ is discrete and sample sizes among each $X$ level are large, the expected benefit of a marker conditional on a particular $X=x$ can be estimated by modeling the disease risk as a function of $Y$ and estimating the distribution of $Y$ [15] within each stratum of $X$ separately. This approach, however, does not use data as efficiently, nor is it applicable when $X$ is continuous. The semiparametric approach, in contrast, provides a way to borrow information across levels of $X$ and applies to general $X$ including both discrete and continuous components.

### Inference

In this section, we develop confidence intervals for the subject-specific expected benefit. Let $\rho _{1x}=P(D=1|T=1,X=x)$ and $\rho _{0x}=P(D=1|T=0,X=x)$ indicate the prevalence of $D$ conditional on $X=x$ with or without treatment, respectively. Under standard regularity conditions (RCs) specified in Appendix, when $\Delta (x)=\rho _{0x}-\rho _{1x} \ne \delta$, $\widehat{\mathrm{Cost}}_x^1(\delta )$ and $\widehat{\mathrm{EB}}_x(\delta )$ are asymptotically normally distributed as stated in Theorems 1 and 2.

#### **Theorem 1**

Assume RCs, when $\delta \ne \rho _{0x}-\rho _{1x}$, $\sqrt{n}\left\{ \widehat{\mathrm{Cost}}_x^1(\delta )-\mathrm{Cost}_x^1(\delta )\right\}$ converges to a mean-zero normal random variable as $n\rightarrow \infty$.

#### **Theorem 2**

Assume RCs, when $\delta \ne \rho _{0x}-\rho _{1x}$, $\sqrt{n}\left\{ \widehat{\mathrm{E{B}}}_x(\delta )-\mathrm{EB}_x(\delta )\right\}$ converges to a mean-zero normal random variable as $n\rightarrow \infty$.

Proofs of Theorems 1 and 2 follow from standard arguments and are sketched in Appendix. We also provide expression for the asymptotic variances of $\widehat{\mathrm{Cost}}_x^1(\delta )$ and $\widehat{\mathrm{EB}}_x(\delta )$ in the Appendix. The asymptotic normality of $\widehat{\mathrm{EB}}_x(\delta )/\widehat{\mathrm{Cost}}_x^1(\delta )$, the estimator of the relative cost reduction, follows from the Delta method when $\delta \ne \rho _{0x}-\rho _{1x}$.

When $\delta =\rho _{0x}-\rho _{1x}$, it can be shown that $\sqrt{n}\Bigg (\left[ \sum _{i=1}^n\hat{\Delta }\{x,Y_i^{\star }(x)\}/n-\delta \right] _{+} -(\rho _{0x}-\rho _{1x}-\delta )_{+}\Bigg )$, a constituent of $\sqrt{n}\left\{ \widehat{\mathrm{Cost}}_x^1(\delta )-\mathrm{Cost}_x^1(\delta )\right\}$ and $\sqrt{n}\big \{ \widehat{\mathrm{E{B}}}_x(\delta )-{\mathrm{EB}}_x(\delta )\big \}$, converges to a mixture of 0 and a truncated normal distribution. As a result, asymptotic normality of $\widehat{\mathrm{EB}}_x(\delta )$, $\widehat{\mathrm{Cost}}_x^1(\delta )$, or $\widehat{\mathrm{EB}}_x(\delta )/\widehat{\mathrm{Cost}}_x^1(\delta )$ no longer holds. Even in the scenario where asymptotic normality of these estimators does hold, we recommend the bootstrap for constructing confidence intervals since computation of asymptotic variances of these estimators requires numerical differentiation.

The foregoing abrupt change in the limiting distribution at $\delta =\rho _{0x}-\rho _{1x}$ signals nonregularity, which is anticipated by the nonsmoothness of the max operator [13, 28]. Thus, standard bootstrap percentile confidence intervals can lead to undercoverage when $\delta \simeq \rho _{0x}-\rho _{1x}$ [8]; to avoid undercoverage, we develop an adaptive bootstrap confidence interval by extending the ideas of [5, 21, 26] Specifically, we use a pretest to determine if $\rho _{0x}-\rho _{1x} \simeq \delta$. If the test rejects, then there is strong evidence that $\delta$ is different from $\rho _{0x}-\rho _{1x}$ and we apply the standard percentile bootstrap; if the test accepts, we use a projection interval formed as the union of bootstrap intervals as described below.

Let $b=1,\ldots , B$ index bootstrap samples drawn from the original data with replacement. We add a superscript $b$, to indicate that a statistic has been computed using a bootstrap sample. We construct an adaptive projection confidence interval as follows. For any $r \in \mathbb {R}$ define $\widehat{\mathrm{EB}}_{x,r}^b(\delta )=\sum _{i=1}^n\left[ \hat{\Delta }^b\left\{ x,Y_i^{\star b}(x)\right\} -\delta \right] _{+}/n-\left[ \sum _{i=1}^n\hat{\Delta }^b\left\{ x,Y_i^{\star b}(x)\right\} /n-\delta \right] \times I(r\ge 0)$, $\widehat{\mathrm{Cost}}_{x,r}^{1b}(\delta ){=\!\!\!}\sum _{i=1}^n\widehat{\mathrm{Risk}}^b\left\{ x,Y_i^{\star b}(x)\right\} /n-\left[ \sum _{i=1}^n\hat{\Delta }\left\{ x,Y_i^{\star b}(x)\right\} /n-\delta \right] \times I(r\ge 0)$. Let $\zeta _{\mathrm{EB}_{x}(\delta ), \kappa }(r)$ and $\zeta _{\mathrm{Cost}_x^{1}(\delta ),\kappa }(r)$ denote $(1-\kappa )\times 100\,\%$ percentile bootstrap confidence intervals formed by taking empirical percentiles of $\widehat{\mathrm{EB}}_{x,r}^{b}(\delta )$ and $\widehat{\mathrm{Cost}}_{x,r}^{1b}(\delta )$ over bootstrap samples, respectively. Let $\Gamma _{x,\alpha }(\delta )$ denote an asymptotically valid $(1-\alpha )\times 100\,\%$ confidence interval for $\rho _{0x} - \rho _{1x} - \delta$. The $(1-\kappa -\alpha )\times 100\,\%$ projection intervals for $\mathrm{EB}_x(\delta )$ and $\mathrm{Cost}_x^{1}(\delta )$ are given respectively by $\bigcup _{r\in \Gamma _{x,\alpha }(\delta )}\zeta _{\mathrm{EB}_x(\delta ),\kappa }(r)$ and $\bigcup _{r\in \Gamma _{x,\alpha }(\delta )}\zeta _{\mathrm{Cost}_x^{1}(\delta ),\kappa }(r)$.

We construct an adaptive bootstrap CI for $\mathrm{EB}_x(\delta )$ or $\mathrm{Cost}_x^1(\delta )$ by constructing a standard 95 % bootstrap percentile interval when $|\hat{\rho }_{0x}-\hat{\rho }_{1x} - \delta | > \tau _n$, and constructing a projection interval described above with $\kappa +\alpha =5\,\%$ otherwise, where $\tau _n$ is a sequence of positive constants satisfying $\tau _n\rightarrow 0$ and $\sqrt{n}\tau _n\rightarrow \infty$. As presented in Theorem 3, this adaptive bootstrap CI will have asymptotic coverage equal to 95 % when $\rho _{0x} - \rho _{1x} \ne \delta$ and be conservative otherwise. In this sense, the interval is adaptive.

#### **Theorem 3**

Let $\tau _n$ be a sequence of positive random variables satisfying $\tau _{n} \rightarrow 0$ and $\sqrt{n}\tau _n \rightarrow \infty$ almost surely as $n\rightarrow \infty$. Define $\mathfrak {A}_{x,\alpha }(\delta ) = \Gamma _{x,\alpha }(\delta )$ if $|\hat{\rho }_{0x}-\hat{\rho }_{1x}-\delta | \le \tau _n$ and $\hat{\rho }_{0x}-\hat{\rho }_{1x}-\delta$ otherwise. Let $P^{b}$ denote probability taken with respect to the bootstrap sampling algorithm, conditional on the observed data. Assume RCs, we have

1. $$P^{b}\left( \mathrm{Cost}^1_x(\delta ) \in \bigcup _{r\in \mathfrak {A}_{x,\alpha }(\delta )}\zeta _{\mathrm{Cost}_x^1(\delta ),\kappa }(r) \right) \ge 1-\alpha -\kappa + o_{p}(1)$$;
2. $$P^{b}\left( \mathrm{EB}_x(\delta ) \in \bigcup _{r\in \mathfrak {A}(c)}\zeta _{\mathrm{EB}_x(\delta ),\kappa }(r) \right) \ge 1-\alpha -\kappa + o_{p}(1)$$,

where for $|\hat{\rho }_{0x}-\hat{\rho }_{1x}-\delta | > \tau _n$, $\bigcup _{r\in \mathfrak {A}_{x,\alpha }(\delta )}\zeta _{\mathrm{Cost}_x^1(\delta ),\kappa }(r)$ and $\bigcup _{r\in \mathfrak {A}(c)}\zeta _{\mathrm{EB}_x(\delta ),\kappa }(r)$ refer to standard $95\,\%$ bootstrap confidence intervals for $\mathrm{Cost}_x^1(\delta )$ and $\mathrm{EB}_x(\delta )$. If $\rho _{0x}-\rho _{1x} \ne \delta$ then the right hand side of the foregoing inequalities can be replaced with equalities. A proof of Theorem 3 is sketched in Appendix.

## Simulation Studies

In this section, we conduct simulation studies to investigate performance of the proposed estimators. Suppose we have a continuous covariate $X$ and a continuous biomarker $Y$ that jointly follow a bivariate normal distribution with mean 0, standard deviation 0.5 each, and correlation 0.2. We consider two simulation settings: one from a randomized trial and the other from a cohort/cross-sectional study. We consider the estimation of $\mathrm{EB}_x(\delta )$ and $\mathrm{EB}_x(\delta )/\mathrm{Cost}_x^1(\delta )$ for $x$ equal to the 25, 50, and 75 % percentiles in the distribution of $X$.

In the first setting, we consider a two-arm 1:1 randomized trial where the risk of a binary disease $D$ conditional on $X$, $Y$ and $T$ follows a linear Probit model $P(D=1|T,X,Y)=\Phi (-0.8-0.4T+0.5X+0.5Y-0.5XT-YT)$. The differences in disease prevalence between untreated and treated ($\rho _{0x}-\rho _{1x}$) conditional on the 25, 50, and 75 % percentiles of $X$ are $0.165-0.129=0.036, 0.219-0.122=0.097$, and $0.280-0.115=0.165$, respectively.

In the second setting, we consider a cohort/cross-sectional study where subjects receive no treatment. Suppose the risk of a binary disease $D$ conditional on $X$ and $Y$ in the cohort follows a linear Probit model $P(D=1|T=0,X,Y)=\Phi (-1.5+2X-3Y)$. Moreover, suppose the treatment under consideration has a relative risk $rr=40\,\%$. The differences in disease prevalence between untreated and treated conditional on the 25, 50, and 75 % percentiles of $X$ are $0.133-0.080=0.053, 0.199-0.119=0.080$, and $0.281-0.168=0.113$, respectively.

In each simulation setting, sample sizes of 200, 500, and 2000 are used. Standard bootstrap and adaptive bootstrap confidence intervals are constructed with 1,000 resamples. For the adaptive CI, we use $\alpha =0.01$ and use the projection interval when $|\hat{\rho }_{0x}-\hat{\rho }_{1x}-\delta | < \widehat{\mathrm{SE}}(\hat{\rho }_{0x}-\hat{\rho }_{1x})\times \mathrm{max}\{n^{0.05},\Phi ^{-1}(0.975)\}$, where $\Phi ^{-1}$ is the quantile of the standard normal distribution and $\widehat{\mathrm{SE}}(\hat{\rho }_{0x}-\hat{\rho }_{1x})$ is estimated from a standard bootstrap procedure with the disease risk model and the conditional distribution of $Y$ re-estimated in each resampled data.

Performances of our semiparametric estimators of $\mathrm{EB}_x(\delta )$ and $\mathrm{EB}_x(\delta )/\mathrm{Cost}_x^1(\delta )$ for simulation setting 1 are presented in Tables 1 and 2 for $X$ equal to its 25 and 50 % percentiles. Corresponding results for simulation setting 2 are presented in Tables 3 and 4. In general, our estimators have minimal bias with a sample size as large as 200. Coverage of the percentile bootstrap CI is close to the nominal level when $\delta$ is away from $\rho _{0x}-\rho _{1x}$. An undercoverage, however, is observed for standard percentile bootstrap CI when $\delta$ is close to $\rho _{0x}-\rho _{1x}$, which is not alleviated with the increase of sample size. The adaptive bootstrap CI fixes the undercoverage problem. The same pattern is observed for $X$ equal to its 75 % percentile (results omitted).

**Table 1:** Performance of $\mathrm{EB}_x(\delta )$ estimator based on randomized trial data

	$x_1,\rho _{0x_1}-\rho _{1x_1}=0.036$						$x_2,\rho _{0x_2}-\rho _{1x_2}=0.097$					
$\delta -(\rho _{0x}-\rho _{1x})$	$-0.036$	$-0.016$	$-0.006$	$0$	0.004	0.024	$-0.097$	$-0.027$	$-0.007$	0	0.003	0.023
$\delta$	0	0.02	0.03	0.036	0.04	0.06	0	0.07	0.09	0.097	0.1	0.12
$EB_{x}(\delta )$	0.028	0.036	0.04	0.043	0.042	0.033	0.014	0.035	0.044	0.047	0.046	0.036
$n$	$\mathrm{Bias}\times 100$											
200	$-0.29$	$-0.94$	$-1.37$	$-1.68$	$-1.5$	$-0.68$	0.5	$-0.5$	$-1.25$	$-1.56$	$-1.4$	$-0.59$
500	$-0.04$	$-0.55$	$-0.94$	$-1.26$	$-1.09$	$-0.38$	0.25	$-0.17$	$-0.81$	$-1.12$	$-0.96$	$-0.26$
2,000	0.07	$-0.12$	$-0.4$	$-0.68$	$-0.52$	$-0.03$	0.07	0.03	$-0.32$	$-0.6$	$-0.45$	$-0.01$
	$$\mathrm{SE}\times \sqrt{n}$$											
200	0.28	0.28	0.28	0.28	0.28	0.28	0.25	0.3	0.3	0.3	0.3	0.3
500	0.33	0.33	0.32	0.32	0.32	0.32	0.26	0.34	0.34	0.33	0.33	0.34
2,000	0.4	0.38	0.36	0.36	0.36	0.4	0.27	0.4	0.36	0.36	0.36	0.4
	Coverage of 95 % bootstrap CI											
200	94.42	89.5	84.82	81.34	83.34	91.4	95.84	93.96	88.24	85.12	86.72	93.52
500	95.54	92.32	87.44	82.82	85.62	94.4	95.1	95.34	90.16	86.02	88.34	95.04
2,000	95.56	95.96	91.74	83.58	88.72	96.42	94.44	95.82	93.46	86.38	90.52	96.4
	Coverage of 95 % adaptive bootstrap CI											
200	97.1	97.32	97.06	96.7	96.8	97.24	96	97.84	97.88	97.44	97.54	97.58
500	96.78	97.26	97.36	97.12	97.34	97.32	95.24	97.62	97.98	97.58	97.8	97.42
2,000	95.68	97.18	97.64	97.44	97.8	96.66	94.44	95.98	97.88	97.86	98.24	96.72

Here $x_1$ and $x_2$ correspond to the 25th and the 50th percentiles of $X$, respectively

**Table 2:** Performance of $\mathrm{EB}_x(\delta )/\mathrm{Cost}_x^1(\delta )$ estimator based on randomized trial data

	$x_1,\rho _{0x_1}-\rho _{1x_1}=0.036$						$x_2,\rho _{0x_2}-\rho _{1x_2}=0.097$					
$\delta -(\rho _{0x}-\rho _{1x})$	$-0.036$	$-0.016$	$-0.006$	0	0.004	0.024	$-0.097$	$-0.027$	$-0.007$	0	0.003	0.023
$\delta$	0	0.02	0.03	0.036	0.04	0.06	0	0.07	0.09	0.097	0.1	0.12
$\frac{\mathrm{EB}_x(\delta )}{\mathrm{Cost}_x^1(\delta )}$	0.214	0.24	0.254	0.263	0.252	0.198	0.113	0.183	0.207	0.216	0.208	0.167
$n$	$\mathrm{Bias}\times 100$											
200	$-1.06$	$-4.48$	$-6.45$	$-7.81$	$-7.02$	$-3.26$	3.67	$-1.76$	$-4.57$	$-5.67$	$-5.06$	$-2.1$
500	$-0.17$	$-2.72$	$-4.51$	$-5.88$	$-5.12$	$-1.86$	1.68	$-0.63$	$-3.03$	$-4.12$	$-3.54$	$-1.01$
2,000	0.35	$-0.58$	$-1.84$	$-3.11$	$-2.39$	$-0.21$	0.5	0.16	$-1.14$	$-2.16$	$-1.62$	$-0.06$
	$$\mathrm{SE}\times \sqrt{n}$$											
200	2.05	1.89	1.82	1.77	1.75	1.64	1.84	1.54	1.45	1.42	1.41	1.34
500	2.3	2.07	1.97	1.92	1.89	1.8	1.91	1.7	1.56	1.52	1.51	1.46
2,000	2.6	2.27	2.07	1.99	1.97	2.04	1.97	1.88	1.61	1.56	1.54	1.61
	Coverage of 95 % bootstrap CI											
200	96.54	94.42	92.06	89.96	90.9	94.88	95.48	96.08	92.76	91.14	92.14	95.6
500	96.2	94.38	91.46	88.32	90.14	95.32	95	95.8	92.62	90	91.46	95.4
2,000	95.62	95.8	93.06	87.74	91.1	96.4	94.08	95.64	94.02	88.96	91.82	96.1
	Coverage of 95 % adaptive bootstrap CI											
200	97.58	98.14	98.12	98.12	98.08	98.06	95.62	98.02	98.34	98.24	98.3	98
500	97.28	98.04	98.04	97.92	97.98	97.72	95.28	97.62	98.32	98.2	98.4	97.52
2,000	95.82	97.26	98.08	98.1	98.3	96.94	94.08	95.86	98.18	98.36	98.5	96.74

Here $x_1$ and $x_2$ correspond to the 25th and the 50th percentiles of $X$, respectively

**Table 3:** Performance of $\mathrm{EB}_x(\delta )$ estimator based on cohort data

	$x_1,\rho _{0x_1}-\rho _{1x_1}=0.053$						$x_2,\rho _{0x_2}-\rho _{1x_2}=0.080$					
$\delta -(\rho _{0x}-\rho _{1x})$	$-0.043$	$-0.023$	$-0.003$	$0$	0.007	0.026	$-0.07$	$-0.026$	$-0.01$	0	0	0.02
$\delta$	0.01	0.03	0.05	0.053	0.06	0.08	0.01	0.053	0.07	0.08	0.08	0.1
$\mathrm{EB}_x(\delta )$	0.004	0.016	0.029	0.032	0.03	0.025	0.003	0.025	0.036	0.042	0.042	0.036
$n$	$\mathrm{Bias}\times 100$											
200	0.02	0.04	$-0.28$	$-0.45$	$-0.2$	0.01	0.01	0.06	$-0.07$	$-0.43$	$-0.42$	$-0.01$
500	0.01	0.02	$-0.14$	$-0.29$	$-0.07$	0.01	0	0.02	0	$-0.28$	$-0.27$	0.01
2,000	0	0.01	$-0.03$	$-0.14$	$0$	0.01	0	0.01	0.01	$-0.14$	$-0.13$	0
	$$\mathrm{SE}\times \sqrt{n}$$											
200	0.01	0.03	0.06	0.07	0.09	0.11	0.01	0.05	0.06	0.08	0.08	0.11
500	0.01	0.03	0.06	0.07	0.1	0.11	0.01	0.05	0.06	0.07	0.08	0.12
2000	0.01	0.03	0.05	0.07	0.12	0.11	0.01	0.05	0.06	0.08	0.08	0.12
	Coverage of 95 % bootstrap CI											
200	90.46	97.18	92.18	84.06	95.9	94.5	90.86	95.34	98	87.94	88.78	95.1
500	93.02	94.14	93.8	82.64	96.18	94.72	93.5	93.62	97.98	86.58	87.74	94.76
2,000	94.2	94.38	96.98	81.8	94.48	94.3	94.62	94.48	95.94	85.74	88.34	94.28
	Coverage of 95 % adaptive bootstrap CI											
200	90.42	96.06	97.56	95.84	96.08	94.46	90.86	94.34	98.8	97.08	97.12	95
500	93.02	93.7	98.24	96.26	96.02	94.72	93.5	93.34	98	97.5	97.5	94.76
2,000	94.2	94.38	98.02	96.42	94.32	94.3	94.62	94.48	95.4	96.82	96.9	94.28

Here $x_1$ and $x_2$ correspond to the 25th and the 50th percentiles of $X$, respectively

**Table 4:** Performance of $\mathrm{EB}_x(\delta )/\mathrm{Cost}_x^1(\delta )$ estimator based on cohort data

	$x_1,\rho _{0x_1}-\rho _{1x_1}=0.053$						$x_2,\rho _{0x_2}-\rho _{1x_2}=0.080$					
$\delta -(\rho _{0x}-\rho _{1x})$	$-0.043$	$-0.023$	$-0.003$	0	0.007	0.026	$-0.07$	$-0.026$	$-0.01$	0	0	0.02
$\delta$	0.01	0.03	0.05	0.053	0.06	0.08	0	0.01	0.07	0.08	0.08	0.1
$\frac{\mathrm{EB}_x(\delta )}{\mathrm{Cost}_x^1(\delta )}$	0.045	0.143	0.225	0.238	0.225	0.189	0.023	0.146	0.188	0.211	0.211	0.18
$n$	$\mathrm{Bias}\times 100$											
200	0.59	1.1	$-1.16$	$-2.18$	$-0.96$	$-0.02$	0.22	0.76	0.12	$-1.45$	$-1.42$	0.01
500	0.21	0.43	$-0.59$	$-1.49$	$-0.38$	0	0.07	0.28	0.16	$-1$	$-0.96$	0.03
2,000	0.06	0.12	$-0.1$	$-0.78$	$-0.02$	0.02	0.01	0.08	0.09	$-0.52$	$-0.49$	0.02
	$$\mathrm{SE}\times \sqrt{n}$$											
200	0.29	0.61	0.46	0.41	0.35	0.37	0.12	0.47	0.41	0.32	0.32	0.3
500	0.25	0.6	0.51	0.42	0.33	0.36	0.11	0.45	0.47	0.32	0.32	0.29
2000	0.24	0.58	0.63	0.45	0.33	0.36	0.11	0.45	0.52	0.33	0.33	0.29
	Coverage of 95 % bootstrap CI											
200	92.18	92.94	97	90.86	96.42	96.64	91.88	92.7	96.76	92.76	92.9	96.36
500	93.98	93.9	97.64	88.06	96.48	95.26	93.82	93.92	95.7	90.88	91.1	95.5
2,000	94.48	94.3	96.74	87	96.64	94.4	94.5	94.38	94.36	89.3	90.08	94.4
	Coverage of 95 % adaptive bootstrap CI											
200	92.18	92.76	98.16	98.22	98.58	95.96	91.88	92.62	96.26	98.52	98.64	96.2
500	93.98	93.9	98.1	98.02	98.04	95.06	93.82	93.92	95.12	98.52	98.5	95.08
2,000	94.48	94.3	96.18	97.6	96.6	94.4	94.5	94.38	94.36	97.76	97.82	94.4

Here $x_1$ and $x_2$ correspond to the 25th and the 50th percentiles of $X$, respectively

## Example

We illustrate the methodology by evaluating serum creatinine as a risk prediction marker for renal artery stenosis in hypertensive patients from a cohort study. The original cohort consists of 426 hypertensive patients undergoing renal angiography [19, 20]. Baseline risk is modeled with age, smoking status (ever versus never), and their interaction, gender, recent onset of hypertension, body mass index (BMI), abdominal bruit, atherosclerosis disease, and hypercholesterolaemia.

Suppose an individual patient needs to determine whether measuring serum creatinine is useful in guiding the treatment of renal artery stenosis. Here, for illustrative purposes, we assume the presence of a treatment that can lead to a 30 % reduction in the risk of stenosis. The treatment also has additional costs associated with it. An example of the treatment could be the renal angioplasty, which can stabilize a patient’s renal function but has the potential risk of causing bleeding, additional kidney damage, and total renal failure. The value of serum creatinine in guiding the choice of therapy differs across individuals because of differences in individual patients’ baseline risk, distribution of serum creatinine conditional on baseline covariates, and tolerance about treatment harm relative to disease harm. To illustrate the personalized benefit of serum creatinine, we display the subject-specific cost and expected benefit for two individuals using the method described in Sect. 2.1.2. We model the risk of stenosis conditional on baseline covariates and serum creatinine using a linear logistic model, and model the distribution of serum creatinine conditional on baseline covariates with a location-scale model. For the latter, a multiple linear regression model is used for the location parameter conditional on baseline covariates, and a constant scale parameter is assumed. Subject 1 is a 33-year-old male with BMI 19.9 kg $\mathrm{m^{-2}}$ who has a smoking history. His baseline risk of renal stenosis is 15.7 %. Subject 2 is a 65-year-old female with BMI 26.0 kg $\mathrm{m^{-2}}$ who has vascular diseases. Her baseline risk is 38.3 %.

Figure 1a shows the minimum total disease and treatment cost for each subject as a function of the cost ratio $\delta$ with or without the serum creatinine measurement. Corresponding subject-specific expected benefit curves are shown in Fig. 1b, and the curves of the relative reduction in cost by measuring serum creatinine are shown in Fig. 1c. These curves provide useful information to an individual for determining whether to have the new marker measured. For example, if subject 2 has a high tolerance for treatment cost with a cost ratio $\delta =5\,\%$, she will have an expected benefit of 1e$-$4 [with 95 % CI (0, 0.005)] and a relative cost reduction of 0.04 % [95 % CI (0–2.6 %)] by measuring serum creatinine, which might be deemed too minimal to justify the measurement of the marker. In contrast, if subject 1 has the same cost ratio of $5\,\%$, his expected benefit will be 0.007 [95 % CI (4e$-$4, 0.068)], amounting to a relative cost reduction of 5.2 % [95 % CI (0.2, 32.6 %)], which may be large enough for him to choose the marker measure. On the other hand, suppose subject 1 has a low tolerance for treatment cost with $\delta =15\,\%$, his expected benefit will decrease to 0.002 [95 % CI (0, 0.012)], which corresponds to 1.3 % reduction in cost [95 % CI (0, 4.3 %)] by measuring serum creatinine. At this cost ratio, subject 2 will have a higher expected benefit of 0.022 [95 % CI (0.002, 0.083)] with a relative cost reduction of 5.9 % [95 % CI (0.5, 26.7 %)].

In practice, given an individual’s baseline covariates, the subject-specific expected benefit curves can be generated. The individual can then find out the value of measuring the additional marker by inputting his/her choice of the cost ratio $\delta$ or a range of $\delta$, to assist with the decision regarding the diagnostic test.

## Concluding Remarks

A biomarker that is useful in guiding treatment decisions for the general population will have different values to individual patients due to individual differences in their responses to treatment and in their tolerances of the disease harm and treatment harm. In this paper, we propose new graphical tools for personalized evaluation of a biomarker’s value in guiding clinical decisions from a cost-benefit perspective. In particular, based on an individual’s baseline covariate level and personal input of treatment/disease cost ratio, we propose to evaluate the biomarker-elicitated reduction in total disease and treatment cost, assuming a person will make the most cost-effective treatment decision before and after measuring the biomarker. Besides the absolute reduction in cost, the relative reduction is also recommended to help with personal decision making. In practice, a subject can compare the expected benefit of a biomarker with the cost of measuring the marker to make a final judgment call about whether to have the biomarker measured.

A caveat in cost-benefit analyses at a population level is the choice of the treatment/disease cost ratio, which is typically difficult to fix across individuals. Different approaches have been taken to reach a common cost ratio. For example, in the setting where treatment harm is characterized by other diseases caused by the treatment, one can estimate the cost ratio using data on different types of disease outcomes, under an assumption about the relative severity between the targeted disease events and the side effect of treatment [10]; other researchers have proposed to elicit a common cost ratio across subjects using a questionnaire [30]. For personalized evaluation of a biomarker, on the other hand, allowing for subject-specific input of the cost ratio is important in order to flexibly account for individual differences. The expected benefit conditional on covariates and a personal input $\delta$ essentially quantifies the value of measuring the marker to individuals in the population who have the same potential disease risk and cost ratio as this particular subject.

Estimators for our proposed measures were derived both in a randomized trial setting where the disease risk is modeled as a function of the marker, the covariates, and the treatment, and in a cohort study setting where the disease risk model in the absence of treatment is modeled as a function of the marker and the covariates. An adaptive bootstrap procedure was developed for inference to account for nonsmoothness in the estimands.

While described in a personalized decision setting, the methods we developed can be readily applied to evaluate the value of biomarkers in treatment decision making among sub-populations, which can differ with respect to baseline covariates and cost ratio.

## Appendix

### Proof of Theorems 1 and 2

Let $\theta$ be the parameters for GLM model: $P(D=1|T,X,Y)=G(T,X,Y;\theta )$ (for a randomized trial) or $P(D=1|T=0,X,Y)=G(X,Y;\theta )$ for a cohort/cross-sectional study. Denote $\mathrm{Risk}(X,Y)=P(D=1|T=0,X,Y)$ as $\mathrm{Risk}(X,Y;\theta )$, $\Delta (X,Y)=P(D=1|T=0,X,Y)-P(D=1|T=1,X,Y)$ as $\Delta (X,Y;\theta )$. Let $\hat{\theta }$ be the estimate of $\theta$, we make the following assumptions:

**Assumptions**

(i) $\sqrt{n}(\hat{\theta }_n-\theta _0 )=n^{-\frac{1}{2}}\sum _{i=1}^{n}\Psi _{2i}+o_{p}(1)$, where $\Psi _{2i},i=1,\ldots , n$ are independently identically distributed variables with $E(\Psi _{2i}|Y_{i},X_i)=0$ and $\mathrm{var}(\Psi _{2i})\equiv \sigma ^2(\theta _0)$.
(ii) $\mathrm{Risk}(X,Y;s)$ is differentiable at $s=\theta$, $X=x$, and $Y=y \in {\mathbb R}$ for almost all $x,y$.
(iii) $\Delta (X,Y;s)$ is differentiable at $s=\theta$, $X=x$, and $Y=y \in {\mathbb R}$ for almost all $x,y$.
(iv) $F_0(.)$ is differentiable everywhere with positive derivative $f_0$.
(v) $E\left[ \left\{ \Delta (X,Y;s)-\delta \right\} _{+}|X\right]$ is differentiable with respect to $s$ at $s=\theta$, $X=x$ for almost all $x$.
(vi) $E\left\{ \left( \Delta \left[ x,{\left\{ Y-s^{\prime }U(X)\right\} }\times \frac{\exp \left\{ t^{\prime }W(x)\right\} }{\exp \left\{ t^{\prime }W(X)\right\} }+s^{\prime }U(x);\theta \right] -\delta \right) _{+}\right\}$ is differentiable with respect to $s$ and $t$ at $s=\gamma$, $t=\eta$.

Let $\hat{\gamma }$, $\hat{\eta }$ be the solutions to

$$\begin{aligned} \sum _{i=1}^n U_{i}(Y_i-\gamma ^{\prime }U_{i})/\sigma _X^2=0, \end{aligned}$$

and

$$\begin{aligned} \sum _{i=1}^n W_{i}\left[ \left( Y_i-\gamma ^{\prime }U_{i}\right) ^2-\sigma _X^2\right] /\sigma _X^2=0. \end{aligned}$$

Then, through Taylor expansion,

$\sqrt{n}\left( \hat{\gamma }-\gamma \right) \simeq \frac{1}{\sqrt{n}}\mathbb {X}_{1}^{-1}\sum _{i=1}^n U_i(Y_i-\gamma ^{\prime } U_i)/\mathrm{e}^{2\eta ^{\prime }W_i}$, where $\mathbb {X}_1$ is limit of $\bar{X}_1=\frac{1}{n}\sum _{i=1}^n U_iU_i^T/\mathrm{e}^{2\eta ^{\prime }W_i}$, and $\sqrt{n}\left( \hat{\eta }-\eta \right) {\simeq }\frac{1}{\sqrt{n}}\mathbb {X}_2^{-1}\sum _{i=1}^n W_i\left[ \left( Y_i-\gamma ^{\prime }U_i\right) ^2{-}\mathrm{e}^{2\eta ^{\prime }W_i}\right] /\mathrm{e}^{2\eta ^{\prime }W_i}$, where $\mathbb {X}_2$ is limit of $\bar{X}_2=\frac{1}{n}\sum _{i=1}^n 2W_iW_i^T\left( Y_i-\gamma ^{\prime }U_i\right) ^2/\mathrm{e}^{2\eta ^{\prime }W_i}$.

By the standard central limit theorem, $\sqrt{n}\left( \hat{\gamma }-\gamma \right)$ and $\sqrt{n}\left( \hat{\eta }-\eta \right)$ converges in distribution to a mean 0 multivariate normal random variable.

Denote $\hat{F}_0(c)=\frac{1}{n}\sum _{i=1}^nI\left( \frac{Y_i-\hat{\gamma }^{\prime }U_i}{\mathrm{e}^{\hat{\eta }^{\prime }W_i}}\le c\right)$, $\hat{F}_X(c)=\hat{F}_0(\frac{c-\hat{\gamma }^{\prime }U}{e^{\hat{\eta }^{\prime }W}})$, then $\hat{F}_X^{-1}(v)=\hat{F}_0^{-1}(v)\times e^{\hat{\eta }'W}+\hat{\gamma }^{\prime }U$ for $v \in (0,1)$.

By functional central limit theorem [24] that for any $\eta >0$, $\frac{1}{\sqrt{n}}\sum _{i=1}^n \left[ I(\left( \frac{Y_i-{\gamma ^*}'U_i}{e^{{\eta ^*}'W_i}}\le c\right) -F_0\left( \frac{ce^{{\eta ^*}'W_i}+(\gamma ^*-\gamma )'U_i}{e^{\eta 'W_i}}\right) \right]$, $(c,\gamma ^*,\eta ^*)\in [F_0^{-1}(a), F_0^{-1}(b)]\times D_{\eta }^{\gamma ^*,\eta ^*}$, converges in distribution to a Gaussian process, where $D_{\eta }^{\gamma ^*,\eta ^*}=\left\{ \gamma ^*,\eta ^*:\Vert (\gamma ^*,\eta ^*)^T-(\gamma ,\eta )\Vert \le \eta \right\}$, for $0<a<b<1$. It then follows from the equicontinuity [6, 24] of the foregoing process and the consistency of $\hat{\gamma }$ and $\hat{\eta }$ that

$$\begin{aligned} \mathrm{sup}_{c\in [F_0^{-1}(a),F_0^{-1}(b)]}&\left| \frac{1}{\sqrt{n}}\sum _{i=1}^n\left[ I\left( \frac{Y_i-\hat{\gamma }'U_i}{\mathrm{e}^{\hat{\eta }'W_i}}\le c\right) -F_0\left\{ \frac{c\mathrm{e}^{\hat{\eta }'W_i+(\hat{\gamma }-\gamma )'U_i}}{\mathrm{e}^{\eta 'W_i}}\right\} \right. \right. \\&- \left. \frac{1}{\sqrt{n}}\sum _{i=1}^n\left[ I\left( \frac{Y_i-\gamma 'U_i}{\mathrm{e}^{\eta 'W_i}}\le c\right) -F_0(c)\right] \right| \rightarrow 0 \end{aligned}$$

in probability. Thus,

$$\begin{aligned}&\sqrt{n}\left\{ \hat{F}_0(c)-F_0(c)\right\} \\&\simeq \frac{1}{\sqrt{n}}\sum _{i=1}^n\left[ I\left( \frac{Y_i-\gamma 'U_i}{\mathrm{e}^{\eta 'W_i}}\le c\right) -F_0(c)+ F_0\left\{ \frac{c\mathrm{e}^{\hat{\eta }'W_i+(\hat{\gamma }-\gamma )'U_i}}{\mathrm{e}^{\eta 'W_i}}\right\} -F_0(c)\right] \\&\simeq \frac{1}{\sqrt{n}}\sum _{i=1}^n\left[ I\left( \frac{Y_i-\gamma 'U_i}{\mathrm{e}^{\eta 'W_i}}\le c\right) -F_0(c)+f_0(c)\left\{ \frac{U_i'}{\mathrm{e}^{\eta 'W_i}}(\hat{\gamma }-\gamma )+cW_i'(\hat{\eta }-\eta )\right\} \right] \\&\simeq \frac{1}{\sqrt{n}}\sum _{i=1}^n\left[ I\left( \frac{Y_i-\gamma 'U_i}{\mathrm{e}^{\eta 'W_i}}\le c\right) -F_0(c)+f_0(c)\left\{ (\mathbb {X}_3'\mathbb {X}_1^{-1}U_i)(Y_i-\gamma ' U_i)/\mathrm{e}^{2\eta 'W_i}\right. \right. \\&+\left. \left. c(\mathbb {X}_4'\mathbb {X}_2^{-1}W_i)\left[ \left( Y_i-\gamma 'U_i\right) ^2-\mathrm{e}^{2\eta 'W_i}\right] /\mathrm{e}^{2\eta 'W_i}\right\} \right] , \end{aligned}$$

where $\mathbb {X}_3$ is the limit of $\bar{X}_3=\sum _{i=1}^n\frac{U_i}{\mathrm{e}^{\eta 'W_i}}/n$, and $\mathbb {X}_4$ is the limit of $\bar{X}_4=\sum _{i=1}^nW_i/n$.

From the functional central limit theorem, we see that $\sqrt{n}\left\{ \hat{F}_0(c)-F_0(c)\right\}$ converges in distribution to a mean 0 Gaussian process. It then follows from a Taylor series expansion and the stochastic equicontinuity of $\sqrt{n}\left\{ \hat{F}_0(c)-F_0(c)\right\}$ that

$$\begin{aligned}&\sqrt{n}\left[ \left\{ \hat{F}_x^{-1}(v)\right\} -F_x^{-1}(v)\right] \\&= \sqrt{n}\left[ \left( \hat{F}_0^{-1}(v)\times \mathrm{e}^{\hat{\eta }^{\prime }w}+\hat{\gamma }^{\prime }u\right) -F_x^{-1}(v)\right] \\&= \sqrt{n}\mathrm{e}^{\eta ^{\prime }w}\left\{ \hat{F}_0^{-1}(s)-F_0^{-1}(s)\right\} +\sqrt{n}F_0^{-1}(s)\mathrm{e}^{\eta ^{\prime }w}w(\hat{\eta }-\eta )+\sqrt{n}u(\hat{\gamma }-\gamma )\\&\simeq -\frac{1}{\sqrt{n}}\frac{\mathrm{e}^{\eta ^{\prime }w}}{f_0(F_0^{-1}(s))}\sum _{i=1}^n\left[ I\left( \frac{Y_i-\gamma ^{\prime }U_i}{\mathrm{e}^{\eta ^{\prime }W_i}}\le F_0^{-1}(v)\right) -v\right. +f_0(F_0^{-1}(v))\\&\times \left. \left\{ \frac{(\mathbb {X}_3-u)'(\mathbb {X}_1^{-1}U_i)(Y_i-\gamma ^{\prime } U_i)}{e^{2\eta 'W_i}}\right. \right. \\&+\left. \left. F_0^{-1}(v)\frac{(\mathbb {X}_4-F_0^{-1}(s)w)'(\mathbb {X}_2^{-1}W_i)\left[ \left( Y_i-\gamma ^{\prime }U_i\right) ^2-\mathrm{e}^{2\eta 'W_i}\right] }{\mathrm{e}^{2\eta 'W_i}}\right\} \right] . \end{aligned}$$

(i) For estimation of $\mathrm{Cost}_{x}^1(\delta )$, we have

$$\begin{aligned}&\sqrt{n}\left( \widehat{\mathrm{Cost}}_x^1-\mathrm{Cost}_x^1(\delta )\right) \\&= \sqrt{n}\left[ \frac{1}{n}\sum _{i=1}^n\widehat{\mathrm{Risk}}(x,Y_i^{\star }(x)) -\left\{ \frac{1}{n}\sum _{i=1}^n\hat{\Delta }(x,Y_i^{\star }(x))-\delta \right\} _{+}\right] \\&-\sqrt{n}\left[ \rho _{0x}-\left\{ \Delta (x)-\delta \right\} _{+}\right] = A-B, \end{aligned}$$

where,

$$\begin{aligned} A&= \sqrt{n}\left\{ \frac{1}{n}\sum _{i=1}^n\widehat{\mathrm{Risk}}(x,Y_i^{\star }(x))-E_{Y|x}\{E(D|X=x,T=0,Y)\}\right\} \\&= \sqrt{n}\left\{ \frac{1}{n}\sum _{i=1}^n\widehat{\mathrm{Risk}}(x,Y_i^{\star }(x))-\frac{1}{n}\sum _{i=1}^n\mathrm{Risk}(x,Y_i^{\star }(x))\right\} \\&+\sqrt{n}\left\{ \frac{1}{n}\sum _{i=1}^n\mathrm{Risk}(x,Y_i^{\star }(x))-E_{Y|x}\left\{ E(D|X=x,T=0,Y)\right\} \right\} \\&\simeq \sqrt{n}\left[ \int \widehat{\mathrm{Risk}}\left\{ x,F_x^{-1}(s)\right\} ds-\int \mathrm{Risk}\left\{ x,F_x^{-1}(s)\right\} \mathrm{d}s\right] \\&+\sqrt{n}\left[ \int \mathrm{Risk}\left\{ x,\hat{F}_x^{-1}(s)\right\} ds-\int \mathrm{Risk}\left\{ x,F_x^{-1}(s)\right\} \mathrm{d}s\right] \\&\simeq \int \frac{\partial \mathrm{Risk}\left\{ x,F^{-1}_x(s),\theta \right\} }{\partial \theta }\sqrt{n}(\hat{\theta }-\theta )\mathrm{d}s\\&+\int \frac{\partial \mathrm{Risk}(x,y)}{\partial y}|_{y=F_x^{-1}(s)}\sqrt{n}\left\{ \hat{F}_x^{-1}(s)-F_x^{-1}(s)\right\} \mathrm{d}s, \end{aligned}$$

$$\begin{aligned} B&= \sqrt{n}\left[ \left\{ \frac{1}{n}\sum _{i=1}^n\hat{\Delta }(x,Y_i^{\star }(x)) -\delta \right\} \times I\left\{ \hat{\Delta }(x,Y_i^{\star }(x))>\delta \right\} \right. \\&-\left. \left\{ \Delta (x)-\delta \right\} \times I\left\{ \Delta (x)>\delta \right\} \right] \end{aligned}$$

which when $$\Delta (x)\ne \delta $$

$$\begin{aligned}&\simeq I\left\{ \Delta (x)>\delta \right\} \sqrt{n}\left\{ \frac{1}{n}\sum _{i=1}^n \hat{\Delta }(x,Y_i^{\star }(x))-\Delta (x)\right\} \\&\simeq I\left\{ \Delta (x)>\delta \right\} \times \sqrt{n}\left[ \int \Delta (x,F^{-1}_x(s))ds-\int \Delta (x,F_x^{-1}(s))\mathrm{d}s\right] \\&\simeq I\left\{ \Delta (x)>\delta \right\} \times \int \frac{\partial \Delta (x,y)}{\partial (y)}|_{y=F_x^{-1}(s)}\times \sqrt{n}\left\{ \hat{F}_x^{-1}(s)-F_x^{-1}(s)\right\} \mathrm{d}s. \end{aligned}$$

(ii) For estimation of $\mathrm{EB}_x(\delta )$, we have

$$\begin{aligned}&\sqrt{n}\left( \widehat{\mathrm{EB}}_x(\delta )-\mathrm{EB}_x(\delta )\right) \\&= \sqrt{n}\left[ \frac{1}{n}\sum _{i=1}^n\left\{ \widehat{\Delta }(x,Y_i^{\star }(x))-\delta \right\} _{+}-\left\{ \frac{1}{n}\sum _{i=1}^n\hat{\Delta }(x,Y_i^{\star }(x))-\delta \right\} _{+}\right] \\&-\sqrt{n}\left( E\left[ \{\Delta (X,Y)-\delta \}_{+}|X=x\right] -\left\{ \Delta (x)-\delta \right\} _{+}\right) \\&= C-B \end{aligned}$$

where $C=\sqrt{n}\left\{ {\mathbb {P}}_n g(x,X,Y,\hat{\gamma },\hat{\theta },\hat{\eta })-Pg(x,Y,\theta ,\gamma ,\eta )\right\}$, with $g$ defined as

$$\begin{aligned} g(x,X,Y,\theta ,\gamma ,\eta )=\left\{ \Delta (x,\gamma ^{\prime }u(x)+\exp \left\{ \eta ^{\prime }w(x)\right\} \frac{Y-\gamma ^{\prime }U(X)}{\exp (\eta ^{\prime }W(X))}-\delta \right\} _{+}. \end{aligned}$$

Note that $\mathcal{{G}}=\left\{ g(\cdot ,\cdot ,\cdot ,\theta ,\gamma ,\eta \}:\theta \in {\mathbb {R}},\gamma \in {\mathbb {R}},\eta \in {\mathbb {R}}\right\}$ is Donsker. Thus equicontinuity follows. In particular,

$$\begin{aligned} C&= \sqrt{n}\left[ \frac{1}{n}\sum _{i=1}^n\left\{ \widehat{\Delta }(x,Y_i^{\star }(x))-\delta \right\} _{+}-E_{Y|x}\{\Delta (x,Y)-\delta \}_{+}\right] \\&= \sqrt{n}\left( \int \left[ \hat{\Delta }\left\{ x,\hat{F}_x^{-1}(s)\right\} -\delta \right] _{+}\mathrm{d}s-\int \left[ \Delta \left\{ x,\hat{F}_x^{-1}(s)\right\} -\delta \right] _{+}\mathrm{d}s\right) \\&+\sqrt{n}\left[ \int \left[ \Delta \left\{ x,\hat{F}_x^{-1}(s)\right\} -\delta \right] _{+}\mathrm{d}s-\int \left[ \Delta \left\{ x,F_x^{-1}(s)\right\} -\delta \right] _{+}\mathrm{d}s\right] \\&\simeq \sqrt{n}\left( \int \left[ \hat{\Delta }\left\{ x,F_x^{-1}(s)\right\} -\delta \right] _{+}\mathrm{d}s-\int \left[ \Delta \left\{ x,F_x^{-1}(s)\right\} -\delta \right] _{+}\mathrm{d}s\right) \\&+\sqrt{n}\left\{ \frac{1}{n}\sum _{i=1}^n\left\{ \Delta (x,Y_i^{\star }(x))-\delta \right\} _{+}\right\} -\sqrt{n}\left\{ \frac{1}{n}\sum _{i=1}^n\left\{ \Delta (x,Y_i^{\dag }(x))-\delta \right\} _{+}\right\} \\&+\sqrt{n}\left\{ \frac{1}{n}\sum _{i=1}^n\left\{ \Delta (x,Y_i^{\dag }(x))-\delta \right\} _{+}-E_{Y|x}\left\{ \Delta (x,Y)-\delta \right\} _{+}\right\} \\&\simeq \frac{\partial \int \left[ \Delta \left\{ x,F^{-1}_x(s),\theta \right\} -\delta \right] _{+}\mathrm{d}s}{\partial \theta }\sqrt{n} \left( \hat{\theta }-\theta \right) \\&+\left( \frac{\partial K}{\partial \gamma },\frac{\partial K}{\partial \eta }\right) \sqrt{n}\left\{ \left( \begin{array}{c}\hat{\gamma }\\ \hat{\eta }\end{array}\right) - \left( \begin{array}{c}{\gamma }\\ {\eta }\end{array}\right) \right\} \\&+\sqrt{n}\left[ \frac{1}{n}\sum _{i=1}^n\left\{ \Delta (x,Y_i^{\dag }(x))-\delta \right\} _{+}-E_{Y|x}\left\{ \Delta (x,Y)-\delta \right\} _{+}\right] , \end{aligned}$$

where

$$\begin{aligned}&K(x,\gamma ,\eta ,\theta )=\\&E\left\{ \left( \Delta \left[ U(x),\frac{Y-\gamma ^{\prime }U(X)}{\exp \{\eta ^{\prime }W(X)\}}\times \exp \{\eta ^{\prime }W(x)\}+\gamma ^{\prime }U(x);\theta \right] -\delta \right) _{+}\right\} , \end{aligned}$$

and $$Y^{\dag }_i(x)={\gamma }^{\prime }U(x)+\exp \left\{ {\eta }^{\prime }W(x)\right\} \times \left\{ Y_i-{\gamma }^{\prime }U(X_i)\right\} /\exp \left\{ {\eta }^{\prime }W(X_i)\right\} $$.

### Proof of Theorem 3

We sketch the proof for the results for $\mathrm{EB}_x(\delta )$, the proof for $\mathrm{Cost}_x^1(\delta )$ is similar.

Define $\mathrm{EB}_{x,r}(\delta )=E\left[ \left\{ \Delta (X,Y)-\delta \right\} _{+}|X=x\right] -\left\{ \Delta (x)-\delta \right\} I(r\ge 0)$. Then following similar arguments as in [15], one can show that $\sqrt{n}\left\{ \widehat{\mathrm{EB}}_{x,r}(\delta )-\mathrm{EB}_{x,r}(\delta )\right\}$ and $\sqrt{n}\left\{ \widehat{\mathrm{EB}}^b_{x,r}(\delta )-\widehat{\mathrm{EB}}_{x,r}(\delta )\right\}$ converge to the same limiting distribution in probability. Thus (i) the validity of the projection confidence intervals when $\rho _{0x}-\rho _{1x}=\delta$ follows from standard arguments for the validity of projection interval (see, for example, [5]).

Moreover, (ii), when $\rho _{0x}-\rho _{1x}\ne \delta$, the standard bootstrap confidence interval provides the correct coverage.

Suppose $\tau _n$ is a positive sequence of random variables converging to zero almost surely with $n$ and satisfying $\sqrt{n} \tau _n \rightarrow \infty$ almost surely as $n\rightarrow \infty$. Define the event $\mathcal {E} \triangleq \lbrace |\hat{\rho }_{0x} - \hat{\rho }_{1x} - \delta | \le \tau _n \rbrace$ then $1_{\mathcal {E}} \rightarrow 1_{\rho _{0x} - \rho _{1x} = \delta }$ in probability. This, together with Results (i) and (ii) proves the validity of the proposed adaptive bootstrap confidence interval.
